# Quantitative Analysis of Inequality in the Distribution of Health Resources Within the Bulgarian Health System

**DOI:** 10.3390/healthcare14111579

**Published:** 2026-06-04

**Authors:** Nikolay Georgiev Atanasov

**Affiliations:** Department of Health Management and Health Economics, Medical University of Plovdiv, 4002 Plovdiv, Bulgaria; nikolay.atanasov@mu-plovdiv.bg; Tel.: +359-885-771-262

**Keywords:** health inequalities, health resources, territorial allocation, concentration index, Gini coefficient

## Abstract

**Highlights:**

This article investigates health inequalities by analysing the regional distribution of health resources in the Bulgarian health system from 2019 to 2023. The study uses primary groups of medical professionals, hospital beds, and outpatient facilities as variables to assess health resources.

**What are the main findings?**

- The income inequality among administrative regions, measured by the Gini index using GDP per capita data, is estimated at approximately 0.29.
- Substantial concentration of the health resources in regions with higher income per capita, with a concentration index of the absolute quantities of resources varying between 0.43 and 0.5 with a relative index of inequality ranging from 1.32 to 1.98, depending on the resource’s group. The concentration indices of resources density are ranging from 0.042 to 0.126, showing a lower-to-moderate level of inequality across poorer and richest regions.

**What are the implications of the main findings?**

- Taken together, these findings contribute to a better understanding of the geographical allocation of labour and material resources within the Bulgarian health system.
- Ultimately, these results are valuable for informing strategies to improve both territorial and financial accessibility to health services.

**Abstract:**

**Background/Objectives**: One contemporary problem in health economics is the measurement and interpretation of socioeconomic inequalities in outcomes, utilisation, and resource distribution. This article aims to estimate socioeconomic inequality in the regional allocation of health resources in Bulgaria during 2019–2023. **Methods**: A year-by-year database was created. It includes regions (n = 28), population, GDP per capita, and the numbers of practicing physicians, dentists, nurses, midwives, hospital beds, and outpatient facilities. Income inequality is analysed using decile ratios, the Gini coefficient, the Generalised Entropy index, and the Atkinson index. Socioeconomic health inequality is quantified using the concentration index (CI) and the coefficient of variation (CV) of the absolute number and of resource density (per 1000 inhabitants). The socio-economic variable is a regional gross domestic product (GDP) per capita fractional rank and a frequency-weight approach to account for population size is used. The analysis is extended with the relative and slope indices of inequality. The CI of hospital beds, practicing physicians, and nurses is decomposed using the age dependency ratio and the number of hospitalisations by districts. **Results**: The Gini index levels remain stable, with no significant fluctuations, in the narrow range of 29.6–29.7. The highest inequality of the absolute resource’s quantity is among midwives (Mean CI = 0.498, CV = 0.018), and the lowest among nurses (Mean CI = 0.442, CV = 0.024). For material resources, a greater concentration of outpatient organisations in richer areas is observed (Mean CI = 0.481, CV = 0.035) than for hospital beds (Mean CI = 0.427, CV = 0.034). The dynamics and descriptives of inequality of resources’ density follow the same pattern, but with lower average rates, ranging from 0.045 to 0.112. The obtained estimates are statistically significant (*p* < 0.05). The analysis of the regression-based measures confirms, without any doubt, both the magnitude and the direction of the development of inequalities in the territorial distribution of health resources. **Conclusions**: Inequality measures vary by resource group. Significant inequality exists in the distribution of health resources between poorer and richer regions, particularly in material resources, in the outpatient sector. For most resource groups, a very slight decrease in inequality is observed midway through the analysed period. The most significant part of this inequality can be explained by differences in hospital care and income across richer and poorer regions.

## 1. Introduction

Timely and accessible medical services require adequate human, material, and financial resources. The regional allocation of these resources is critical for improving health outcomes. When health resources are distributed unevenly across regions, health outcomes may deteriorate. Such disparities can delay care and reduce system efficiency. Socio-economic health disparities also raise significant ethical concerns, including fairness, social justice, and equitable access to healthcare for all population groups.

Existing literature has examined health-related inequalities in Bulgaria, with a particular focus on socio-economic disparities in healthcare service utilisation and differences by settlement type in the Northeast region. These studies typically employ population surveys and analyse variations in service frequency and utilisation across income groups using odds ratios and Student’s t-tests [1,2]. One aspect of disparities studies is the COVID-19 pandemic. According to Rohova (2022), who has made a significant contribution to this field in Bulgaria, the impact of the pandemic on socio-economic inequality in healthcare service consumption and utilisation remains insufficiently explored [3]. Statistically significant differences in the utilisation of all types of health services, except hospital care, are observed between the lowest and highest income groups (first and fifth quintiles). During the pandemic, access to healthcare services was more severely restricted among disadvantaged groups. Inequality is measured using Student’s t-tests to compare group means, with results presented through Lorenz curves. Furthermore, research on the redistributive effects of household health spending in Bulgaria, using inequality indices, indicates that over time the burden of health costs for low-income households increases and surpasses that of higher-income groups [4].

Several studies have addressed territorial inequalities in resource allocation in Bulgaria. For example, Stoyanova et al. (2019) [5] analyse the territorial distribution of general practitioners and cardiologists from 2010 to 2017 using the Gini index. Their findings indicate low location-based inequality relative to district size, although income or well-being is not used as a rank variable. Cardiologists, as anticipated, are significantly more concentrated in larger settlements [5]. In contrast, Rohova (2017) identifies significant imbalances in the distribution of health personnel [6]. This retrospective analysis, covering 2011 to 2015, measures inequality by year and professional group using Gini coefficients and Lorenz curves, with data presented per 10,000 inhabitants for each region and district. The primary conclusion is that human resources are concentrated in larger regions, particularly among certain specialists. The Lorenz curve is constructed from cumulative percentages of the population and medical professionals. However, the analysis does not incorporate socioeconomic inequality across regions as measured by a welfare variable. Instead, the inequality measures in both Rohova (2017) [6] and Stoyanova et al. (2019) [5] are estimated using cumulative population shares and do not employ decomposition techniques to identify the main contributors to existing disparity.

Despite ongoing scholarly interest, research on territorial socioeconomic health inequalities in Bulgaria remains incomplete. Accordingly, the present study seeks to provide new empirical evidence on inequalities in the distribution of human and material resources across districts, with particular attention to living standards as a general socioeconomic determinant. An attempt is also made to identify the main factors contributing to socioeconomic inequality in resource allocation.

The aim of this study is to quantify regional inequalities in the total numbers and density of health resources using a socioeconomic gradient.

## 2. Materials and Methods

### 2.1. Variables and Data

Publicly available, verifiable data from secondary statistical sources maintained by the National Statistical Institute of the Republic of Bulgaria are used. The compiled variables include region name (n = 28), population, GDP per capita at current prices, and the numbers of practicing physicians, dentists, nurses, midwives, hospital beds, and outpatient facilities. The analysis covers data from 2019 to 2023, enabling a retrospective examination of trends. In total, 10 variables are observed: one economic, two demographic, and seven health-related, across 28 administrative districts over a five-year period. Given the study period and variable count, a 1400-element matrix is constructed.

### 2.2. Statistical Analysis

The methodology employs several measures to assess income and socioeconomic disparities in health. The Gini index, percentile ratios, Generalised Entropy, and Atkinson Indices are employed to evaluate income inequality [7,8,9,10]. The concentration index serves as a principal measure of regional imbalances, reflecting the influence of wealth as a socioeconomic gradient [11,12,13]. The coefficient of variation facilitates comparison of inequality dynamics over time and between groups. To provide a comprehensive assessment of inequalities, concentration indices are calculated using both the absolute values of resource groups and their densities per 1000 inhabitants.

The analysis is also extended by regression-based relative and absolute indices of inequality [14]. This step helps verify the results of concentration indices and provides a more complete interpretation of the processes underlying territorial inequalities. The concentration indices for hospital beds, physicians, and nurses are controlled by regions’ age dependency ratios and the number of hospitalisations. The contribution of the control variables to the inequality is estimated using the Wagstaff, van Doorslaer, and Watanabe (2003) approach [15].

GDP per capita serves as the welfare or socioeconomic rank variable for each administrative region. Population sizes for each region are used as frequency-weighting variables in all regression models, including those used to calculate the concentration index. Relative Index of Inequality (RII) estimates are derived from incidence rate ratios (IRRs) using Poisson and negative binomial regression models, with the logarithm of population size included as an offset variable. The optimal RII estimates are selected based on the Akaike information criterion (AIC) and the Bayesian information criterion (BIC). The Slope Index of Inequality (SII) is calculated from the slope coefficients of simple linear regression models for each health variable, standardised per 1000 inhabitants. Regression estimates are compared across the full sample and samples excluding extreme cases to identify consistent patterns in regional inequalities. Postestimation diagnostics include the deviance goodness-of-fit test, and the variance inflation factor.

All concentration index calculations and statistical analyses are conducted using StataNow version 19.5 SE [16,17,18].

### 2.3. Study Limitations

Secondary statistical sources that encompass the entire society are aggregated, which constitutes an advantage over studies based on observations of specific units, such as survey respondents. Nevertheless, the present study is subject to several notable limitations. A primary limitation is the lack of control over variables, which arises from the nature and scope of the data employed. To address this issue, the methodology of Wagstaff et al. was implemented. Additional limitations include the time lag inherent in the data and the omission of the hidden economy. The study utilises data up to 2023, which precludes consideration of the most recent trends in the variables analysed. This limitation remains unaddressed. One potential solution would be to forecast the variables’ values and extend the analysis to the final year of the period under review. However, the short time frame (2019–2023) restricts the reliability of such forecasts. For GDP and demographic indicators, this is less problematic, as the National Statistical Institute (NSI) provides accessible forecasts for the subsequent year. In contrast, such forecasts are unavailable for health-related variables, including resources, utilisation, and activity volumes. Furthermore, GDP does not account for income or expenditures that are not legally reported, nor does it include outputs from the household economy. These methodological limitations of GDP constrain the understanding of the actual magnitude of public welfare.

## 3. Results

### 3.1. Income Inequality as a Prerequisite for Health Disparities

Health inequalities are shaped by income distribution at the national, regional, and individual levels. Economic development, living standards, and personal income collectively establish the conditions under which health inequalities emerge. The percentile ratios and Gini indices are used to estimate income inequality across regions, as shown in Table 1.

Percentile ratios and Gini index levels exhibit no significant changes during the analysis period. This absence of statistically significant variation is expected, given the improbability of substantial fluctuations in GDP per capita and population size across regions over a relatively short timeframe. Decile ratios indicate that administrative regions with the lowest standard of living have GDP per capita values between 3.9 and 4.2 times lower than those of the wealthiest regions. The corresponding ratio between the richest regions and those at the median income level remains within a narrower range, from 2.78 to 2.89. Notably, the ratio of income at the highest levels to that at the lowest levels is decreasing marginally, suggesting only a minimal trend toward reduced inequality between the richest and poorest regions.

**Figure 1:**
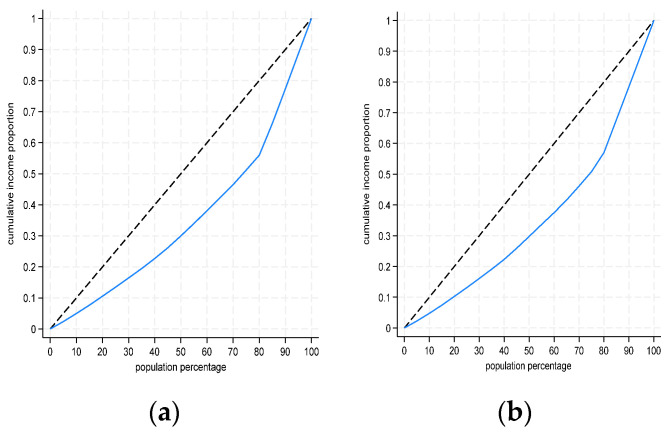
The Lorenz curves for 2019 (**a**) and 2023 (**b**) of GDP per capita by administrative region population.

The Gini indices, which quantify overall inequality, remain nearly constant, ranging from 0.283 to 0.297. The extent of inequality is illustrated by the Lorenz curve in Figure 1.

The slopes of the Lorenz curves at the beginning and end of the period do not differ significantly. Higher- and lower-income regions are farther from the perfect equality line.

In addition to the Gini index, the Generalised Entropy measures indicate which parts of the income distribution are more sensitive to inequality or move further from perfect equality (where all regions have equal GDP per capita). At α = −1, the index’s sensitivity is reflected in the distribution of the lowest incomes, i.e., in areas with the lowest standard of living. In this case, the GE ranges from 0.132 to 0.146, with the highest levels at the beginning and end of the period (Table 2). In other words, at the end of the period, the deviation from perfect equality in the poorest areas has remained almost unchanged and may even have increased slightly (GE = 0.146 in 2023). Across the low-income to average-income range, we observe the same trend. In the above-average-income regions, the index increases significantly. It reaches its maximum levels at α = 2 (GE = 0.196 for 2019). It seems that as income levels increase, the distance from ideal equality widens. Interestingly, a slight easing of inequality is observed in the high-income range in 2023.

The absolute value of the Atkinson index increases annually with the sensitivity parameter for inequality aversion (Table 2). At high income levels (ε = 0), the index ranges from 0.07 to 0.076. This outcome suggests that when societal priorities favour total income over inequality reduction, there is a willingness to forgo between 7% and 7.6% of total income to achieve perfect equality at an equivalent level of social welfare. Conversely, when the sensitivity parameter is higher (ε = 2), indicating a stronger preference for reducing inequality over maximising total income, the index ranges from 0.209 to 0.226. These higher values reflect a substantially greater income sacrifice (20.9% to 22.6%) required to attain egalitarianism while preserving the same level of well-being.

### 3.2. Regional Inequality in Medical Professionals

**Figure 2:**
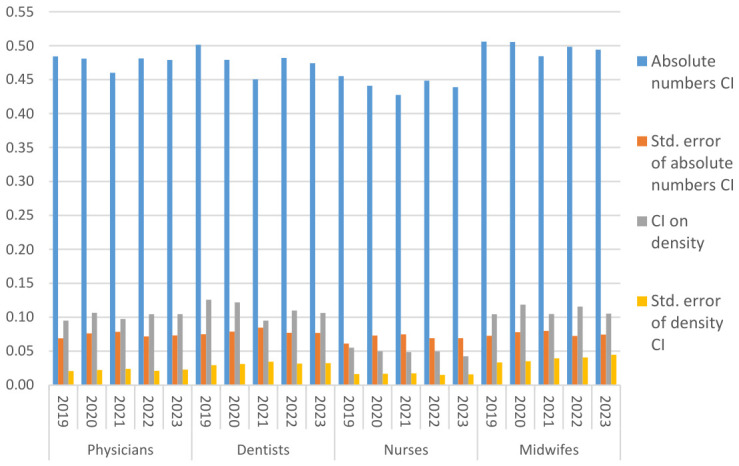
Concentration indices (CI) by main groups of medical professionals.

The primary categories of medical professionals are practicing physicians, nurses, and midwives. Concentration indices for medical professionals, measured by total number, consistently exceed 0.4 (Figure 2). Income inequality indices exhibit similar patterns. Inequality measures peak in the initial year, decline slightly during the middle years, and return to near-initial levels by the end of the period. The highest concentration index is reported for midwives at 0.506 in 2019, closely followed by dental doctors at 0.501 in the same year. The lowest value is observed among nurses in 2021 at 0.427.

This finding accounts for the highest average index levels observed in these two specialist categories. Table 3 presents the average annual indices and their corresponding coefficients of variation.

The groups of nurses and midwives demonstrate the lowest relative temporal fluctuations and the lowest annual average index values. The regional distribution of these groups remains stable over time. In contrast, dentists display the most volatile indicators from 2019 to 2023. Although fluctuations in the concentration index for doctors are relatively low, their absolute averages are among the highest. The concentration of specialists in all four groups is elevated in wealthy administrative districts, with this pattern being particularly pronounced and stable for doctors. Density inequality rates are generally low to moderate and demonstrate a similar descriptive model.

These findings are further supported by indicators of fluctuations among medical specialists. Table 4 presents the annual coefficients of variation for each medical specialist group by district.

The fluctuation in the total number of dentists is the most pronounced, followed by midwives, doctors, and nurses. The coefficients of variation correspond to the levels of the concentration indices and enhance the analysis of territorial inequality as ranked by per capita income.

### 3.3. Regional Inequalities in Material Resources

This study measures material resources using two variables: the number of hospital beds and the number of outpatient facilities. As with medical professionals, these variables are reported both in absolute numbers and per 1000 inhabitants.

**Figure 3:**
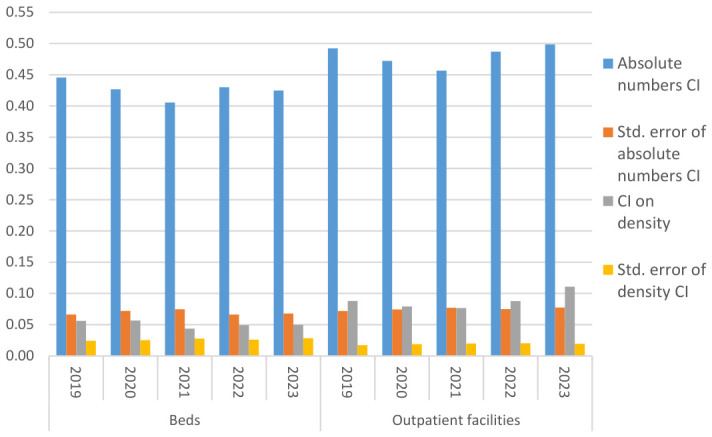
Concentration indices (CI) of hospital beds and outpatient facilities.

Inequality indicators for the total number of physical resources are notably high (Figure 3), with values well above 0.4, mirroring the distribution of human resources. This outcome aligns with expectations, as the rational allocation of medical services requires the development of both physical and human capital in tandem. Consequently, inequality in the distribution of one production factor often leads to similar disparities in other production factors.

There is a pronounced concentration of hospital beds and outpatient services in regions with higher GDP per capita, to the detriment of areas with lower GDP per capita. Density inequality rates reveal moderate disparities and exhibit parallel trends over time. The highest concentration indices are observed for the number and density of outpatient facilities, while inpatient resources display substantially lower inequality rates.

Several specific trends are evident. The concentration indices for hospital beds are marginally lower than those for physicians. The average annual concentration index for outpatient medical organisations is substantially higher (0.481 and 0.089) compared to hospital beds (0.427 and 0.051). The volatility of these indices over time is comparable, with coefficients of variation of 0.035 for non-hospital organisations and 0.034 for hospital beds. An upward trend in inequality is apparent toward the end of the period. Disparities in the distribution of outpatient facilities are particularly pronounced, with these resources concentrated in larger and wealthier districts.

Greater pure inequality is associated with greater temporal variability in the total number of facilities across districts (Table 5). Similarly, the findings of the human resources study are corroborated: higher average annual concentration indices are linked to greater variation in health variables.

### 3.4. Regression-Based Indices of Relative and Absolute Effects

The relative and slope indices of inequality can corroborate or challenge the findings from concentration indices regarding the magnitude of inequality. These indices also enhance understanding of the direction and trend of resource concentration across regions. This is achieved by estimating the regression model’s slopes. Given that health resource data are typically count data, the selection between Poisson and negative binomial regression models is warranted.

Analysis of regression-based indices corroborates the magnitude of existing inequalities identified by the concentration indices (Table 6). The incidence rate ratio (IRR), which measures relative inequality, is consistently well above unity in both regression models. However, the Poisson regression fails the overdispersion test (deviance goodness-of-fit *p*-value < 0.05), while information criteria indicate that the negative binomial regression provides superior explanatory power. Consequently, the IRR from the negative binomial regression is adopted as the measure of relative inequality. At the beginning of the period, the most pronounced relative inequality is observed among dentists, with the richest region (Sofia) having approximately 91% more dentists per capita (IRR = 1.906, 95% CI 1.9046 to 1.907) than the poorest region (Haskovo). By the end of the period, the highest relative inequality shifts to practicing physicians, as the richest region, the capital, exhibits a 94% higher physician provision compared to the poorest region (IRR = 1.944, 95% CI 1.9432 to 1.9454). In contrast, inequality among nurses is the least severe, with the richest regions being 30% to 40% more likely to have nurses than the poorest. The distribution of midwives is higher than that of nurses but lower than that of physicians, particularly at the end of the period. This pattern is primarily attributable to the concentration of larger personnel in maternity wards of major hospitals, which are situated in regions with higher living standards. The disparity in the number of outpatient facilities is the most significant, surpassing inequalities observed in all professional groups. For hospital beds, inequality is more moderate, with richer administrative districts providing 40% to 50% more beds than the poorest districts.

Analysis over time reveals a trend of expanding relative inequalities. This trend is most pronounced among doctors and outpatient health facilities. For these resource groups, the relative inequality indices increase by more than 30 percentage points.

A similar pattern is observed for absolute measures of inequality. For all resource groups except nurses, the SII increases over time. The most significant absolute growth occurs among doctors and hospital beds. At the beginning of the period, achieving absolute equality in the distribution of physicians would require adding 2 doctors per 1000 inhabitants to the poorest region; by the end of the period, 2.8 doctors per 1000 inhabitants are required. Similarly, an additional 2.99 hospital beds per 1000 inhabitants would be needed in the poorest region by the end of the period.

The slope coefficient estimates are highly sensitive to extreme values of the dependent variable. Outliers at the beginning of the period are identified among doctors and nurses in the Pleven district, dentists in the Plovdiv district, and midwives in the Pleven and Varna districts. By the end of the period, this pattern remains largely unchanged, except that the Pleven district reports 12.9 beds per 1000 people, a figure substantially higher than in other regions. The income fractional rank for Pleven in 2023 is 0.5. These regions are notable for hosting medical universities with longstanding histories and strong reputations. Sofia is also recognised as a prominent university centre.

Excluding outliers from the regression analysis consistently increases both the IRR and SII levels. In 2023, the IRR for physicians increases to 2.049 (95% CI 2.048 to 2.05), and the SII reaches 2.998 (95% CI 2.996 to 2.999) per 1000 inhabitants. For hospital beds, the relative inequality is 1.566 (95% CI 1.565 to 1.567), and the absolute index is 3.27 (95% CI 3.266 to 3.274) when the Pleven district is excluded.

Regression-based indices indicate a persistent trend of resource concentration in university cities and wealthier regions, whereas poorer districts continue to lag significantly.

### 3.5. Income as a Key Determinant of Resource Inequality

**Figure 4:**
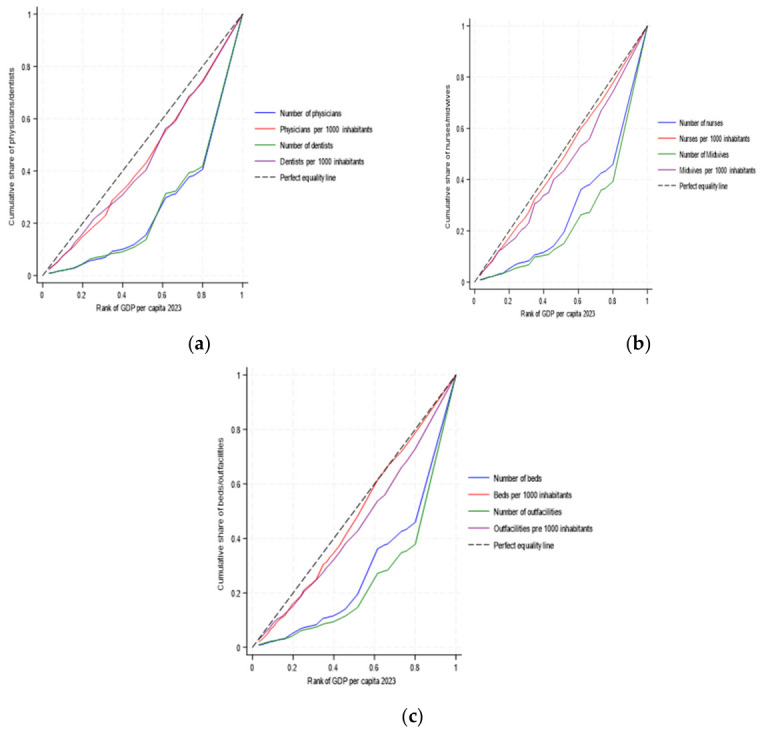
Health resources concentration curves for 2023.

Income inequality and resource inequality display significant parallels, particularly in their persistently elevated rates. The most compelling evidence for this similarity lies in the slopes of the Lorenz and concentration curves, plotted against cumulative population percentages (Figure 1) and GDP per capita (Figure 4). In Figure 4, the concentration curve remains relatively flat at low- to middle-income levels. For income groups above the middle level, the slope increases and exhibits pronounced fluctuations. At the highest income levels, the slope of the concentration curve increases sharply, closely reflecting the pattern observed in the Lorenz curves. In both instances, these changes occur at similar values of the stratifying variable on the *X*-axis.

Stronger evidence in the same direction is also found. Table 7 demonstrates a significant high straightforward relationship between the income variable and the total number of resources, whereas the association with density rates is low to moderate.

Recent evidence demonstrates a pronounced effect of income on disparities in the absolute number of resources. However, this effect does not extend to resource density. Imbalances in total resources largely reflect the pattern of income disparity across districts. Within multivariate models assessing the contribution of various predictors to inequality, the explanatory power of the income variable diminishes.

### 3.6. Decomposition of Concentration Indices of Hospital Beds, Physicians, and Nurses

The relationship between income and concentration indices is assessed while accounting for the age dependency ratio and the number of hospitalisations. Three regression models are constructed for hospital beds, practicing physicians, and nurses, each employing the fractional rank of GDP per capita, the age dependency ratio, and the number of hospitalisations as independent variables. Variance inflation factors for these models remain below 1.5, which supports the assumption that the predictor variables are not highly correlated.

The inclusion of additional control variables alongside income rank substantially increases the models’ explanatory power. The most significant improvement in R2 results from the addition of hospitalisations. This result indicates that hospitalisations represent the most influential specific source of regional inequality, accounting for between 82.4% and 89.3% of the total concentration index (Table 8).

Hospitalisations predominantly occur in the wealthiest regions, where concentration indices exceed 0.49, reflecting significant inequality between affluent and disadvantaged administrative districts. The elasticity coefficients for the hospitalisation variable are also highest in these areas. The greatest elasticity is observed in the physician model for 2023, where a 1% increase in hospitalisations corresponds to a 0.897% increase in the number of practicing physicians. On the other hand, the elasticity below unity may reflect the increasing workload of medical personnel in Bulgarian hospitals.

The demographic control variable exhibits the lowest elasticity coefficient and contributes minimally and negatively to regional inequality, indicating a pro-poor effect. The negative concentration index for the age dependency ratio suggests that the highest proportion of individuals with costly medical needs reside in the poorest districts.

The income rank variable significantly influences regional disparities in resource distribution, with an average elasticity of 0.26. In terms of hospitalisations, their contribution to the total concentration indices remains relatively modest, ranging from 4.06% to 8.26%. This highlights a contradiction between the greater health needs and the limited availability of material and human resources in administrative districts with higher proportions of age-dependent populations and lower income levels.

## 4. Discussion

This study examines the distribution of major health resource groups across Bulgaria’s administrative districts, ranked by per capita income. It identifies the magnitude, patterns, and direction of existing inequalities. The findings demonstrate a clear tendency for health resources to be concentrated in high-income regions, resulting in a corresponding deficit in poorer districts. Quantitative indicators of hospital activity, such as the number of hospitalisations, exert the greatest influence on regional disparities and may have a sustainable impact on medical personnel’s workload.

Previous studies employing similar methodologies have reported lower absolute concentration indices for certain groups of human resources in Bulgaria. For instance, between 2011 and 2015, the Gini indices for physicians ranged from 0.08 to 0.1, while those for nurses ranged from 0.03 to 0.05 [6]. Findings from Stoyanova et al. for general practitioners and cardiologists are consistent with these results [5]. The primary reason for the observed differences is methodological. The studies by Rohova and Stoyanova et al. do not incorporate regional economic status as a variable; instead, they assess inequality across regions solely based on population size. The present findings confirm the concentration of medical specialists and physical resources in more populated regions, which also tend to have higher socioeconomic status.

Bright et al. (2019) found that concentration indices for the distribution of ear, nose, and throat specialists vary significantly across 15 Latin American countries [19]. Some distributional inequality rates are comparable to those observed among medical personnel groups in the present study. However, direct comparisons are limited primarily by differences in data sources and variables. Bright et al. employ the Human Development Index (HDI) as a socio-economic variable, while the current study ranks regions by GDP per capita. Both HDI and the fractional rank of income are bounded between 0 and 1, but HDI is a composite measure that incorporates income, health, and education levels. Additional differences arise from the scope of physician groups analysed. At the beginning of the period, the concentration index (CI) for doctors’ density is 0.0952, rising to 0.105 by the end, indicating an increase in inequality in Bulgaria. Nevertheless, this value remains below the average reported by Bright et al. (RCI = 0.344).

A spatiotemporal study by Shaltynov et al. on Kazakhstan reports findings closely aligned with those observed in the Bulgarian case [20]. The health resource variables and their units are nearly identical in both studies. Socioeconomic ranking is measured by income per capita at current prices, and the dispersion formula is applied to estimate concentration indices. Another notable similarity is that both countries historically maintained highly centralised healthcare systems, with the state exercising strict control over resource allocation across regions. Currently, both systems rely on social security financing for healthcare, and private initiative plays a significant role in each context. A key difference, however, is that in Kazakhstan, the state covers insurance for a broader range of social groups, whereas in Bulgaria, coverage is limited to groups such as pensioners, individuals under 18 years of age, and a few others. Consequently, the state’s contribution to Kazakhstan’s health social fund is substantially larger. These fundamental similarities and differences help explain the processes underlying the distribution of health resources across territories. The present study demonstrates greater similarity with the results of Shaltynov et al. For instance, the Gini index for practicing physicians, while declining after 2016, remains above 0.17 in 2023. In contrast, inequality in hospital bed distribution is lower, with the Gini index fluctuating around 0.1. Both rates exceed the analogous measurements in the current study, indicating a greater imbalance in Kazakhstan. Although direct comparison with outpatient practices is not entirely appropriate, Kazakhstan exhibits a much higher Gini index in primary practices than Bulgaria. A moderate increase in inequality in the territorial distribution of outpatient resources is evident in both countries. The observed trends in equality and territorial disparities in resource provision may be attributed to the liberalisation of the two previously similar health systems.

The findings of this study can be contextualised by referencing the work of Duka et al. (2025) [21], which examines territorial imbalances in nurse density and absolute numbers in Albania. The authors employ Gini indices to assess the distribution of nurses across regions categorised by the HDI. Duka et al. report a Gini index for nurse density of 0.0228, indicating minimal inequality. Despite this low value, the authors highlight concerns regarding disparities between the capital, Tirana, and regions with lower social status. A comparable situation exists in Bulgaria, where Sofia has a substantially higher nurse density (4.72 per 1000 inhabitants) than Haskovo (3.61 per 1000 inhabitants). A higher nurse density concentration index (0.042 in 2023) is observed in Bulgaria, indicating greater regional inequality.

Indicators of health inequalities arise not only from the influence of national economic processes but also from the reimbursement mechanisms applied to healthcare providers. These outcomes are particularly evident within a pay-for-services system. Notably, such payment systems are not designed to identify resource provision imbalances or to implement corrective mechanisms. Additionally, the relative freedom of patient choice contributes to these disparities. When travel distances are not so long, patients often seek consultations and medical care in centres with greater concentrations of human and technological resources. Consequently, access to higher-quality services incurs additional costs for patients, including time and travel expenses. A possible solution outside the payment system would be to increase the role of municipal authorities in order to stimulate local healthcare.

## 5. Conclusions

This study demonstrates that health resources are disproportionately concentrated in regions with higher per capita income, leading to significant disparities compared to areas with lower socioeconomic status. Analysis of overall inequality reveals a pronounced imbalance in health needs and hospital care utilisation between wealthier and poorer regions, highlighting the risks associated with declining financial and geographic access to medical services.

Health authorities should prioritise concentrating and retaining health resources in regions with smaller populations and lower living standards. Policy measures should address both reducing educational costs for nursing, midwifery, and physician assistant qualifications and strengthening smaller municipal hospitals and other local medical facilities. Greater emphasis on the role of local budgets in health spending is warranted. Although current fiscal decentralisation does not grant municipalities substantial revenue autonomy, increasing municipal financial independence could improve the provision of human and material resources within regional healthcare systems.

## Figures and Tables

**Table 1:** Regional income inequality, as measured by percentile ratios and Gini indices, 2019–2023.

	2019	2020	2021	2022	2023
p90/p10	4.198	4.127	3.962	4.119	4.103
p90/p50	2.831	2.890	2.870	2.781	2.789
p10/p50	0.674	0.700	0.724	0.675	0.680
p75/p25	1.634	1.537	1.505	1.744	1.593
Gini	0.297	0.286	0.283	0.294	0.297

**Table 2:** Generalised entropy indices GE(α) and Atkinson indices A(ε).

Index	Income Sensitivity Parameter	2019	2020	2021	2022	2023
GE(α)	α = −1	0.145	0.134	0.132	0.145	0.146
	α = 0	0.148	0.138	0.136	0.142	0.145
	α = 1	0.164	0.154	0.151	0.150	0.156
	α = 2	0.196	0.183	0.180	0.170	0.180
A(ε)	ε = 0.5	0.076	0.071	0.070	0.071	0.073
	ε = 1	0.138	0.129	0.127	0.132	0.135
	ε = 2	0.225	0.212	0.209	0.224	0.226

**Table 3:** Average annual concentration indices and their coefficients of variation (CV) by groups of specialists.

Groups	The Absolute Number of Professionals		Density of Professionals’ Group	
	Mean CI	CV	Mean CI	CV
Physicians	0.477	0.020	0.102	0.049
Dentists	0.477	0.038	0.112	0.111
Nurses	0.442	0.024	0.049	0.092
Midwives	0.498	0.018	0.11	0.062

**Table 4:** Annual coefficients of variation in the number of medical professionals.

Year	Physicians	Dentists	Nurses	Midwives
2019	1.322	1.487	1.231	1.380
2020	1.383	1.481	1.192	1.415
2021	1.372	1.492	1.190	1.404
2022	1.381	1.508	1.203	1.418
2023	1.397	1.504	1.192	1.421

**Table 5:** Annual coefficients of variation in the number of hospital beds and outpatient facilities.

Year	Hospital Beds	Outpatient Facilities
2019	1.1885	1.2995
2020	1.2011	1.2773
2021	1.1921	1.2895
2022	1.1862	1.3642
2023	1.2075	1.4363

**Table 6:** Regression-based inequality indices estimate.

Year	Variable	Poisson Regression		Negative Binomial Regression		Linear Regression
		IRR	AIC	IRR	AIC	SII
2019	Physicians	1.818	3.35 × 10^8^	1.638	9.72 × 10^7^	2.013
	Dentists	2.219	2.29 × 10^8^	1.906	8.26 × 10^7^	0.669
	Nurses	1.502	2.68 × 10^8^	1.366	9.40 × 10^7^	1.309
	Midwives	2.136	1.33 × 10^8^	1.628	7.39 × 10^7^	0.235
	Hospital beds	1.345	7.44 × 10^8^	1.436	1.07 × 10^7^	2.479
	Outpatient facilities	1.869	6.05 × 10^7^	1.658	5.88 × 10^7^	0.129
2023	Physicians	2.007	3.34 × 10^8^	1.944	9.08 × 10^7^	2.811
	Dentists	1.926	2.47 × 10^8^	1.878	7.82 × 10^7^	0.707
	Nurses	1.308	1.83 × 10^8^	1.319	8.53 × 10^7^	1.145
	Midwives	2.275	1.61 × 10^8^	1.849	7.13 × 10^7^	0.303
	Hospital beds	1.242	8.80 × 10^8^	1.505	1.01 × 10^8^	2.993
	Outpatient facilities	2.349	6.14 × 10^7^	1.981	5.74 × 10^7^	0.214

**Table 7:** Correlation coefficients between resources and income per capita for the year 2023.

Groups	Absolute Number of Professionals	Density of the Professionals’ Group
Physicians	0.844	0.473
Dentists	0.795	0.385
Nurses	0.844	0.344
Midwives	0.846	0.263
Hospital beds	0.806	0.240
Outpatient facilities	0.870	0.540

**Table 8:** Decomposition of the concentration indices with income rank and control variables.

Year	Variable	Elasticity ^1^	Concentration Index ^2^	Explained Contribution	
				Absolute	Percentage
2019	*Hospital beds*	-	*0.446*	-	-
	GDP per capita rank	0.115	0.257	0.029	6.599
	Age dependency ratio	0.140	−0.044	−0.006	−1.387
	Hospitalisations	0.771	0.493	0.380	85.284
2023	*Hospital beds*	-	*0.425*	-	-
	GDP per capita rank	0.138	0.255	0.035	8.262
	Age dependency ratio	0.104	−0.044	−0.005	−1.068
	Hospitalisations	0.778	0.477	0.371	87.427
2019	*Physicians*	-	*0.484*	-	-
	GDP per capita rank	0.089	0.257	0.023	4.694
	Age dependency ratio	−0.012	−0.044	0.001	0.112
	Hospitalisations	0.865	0.493	0.426	88.060
2023	*Physicians*	-	*0.479*	-	-
	GDP per capita rank	0.111	0.255	0.028	5.889
	Age dependency ratio	−0.162	−0.044	0.007	1.480
	Hospitalisations	0.897	0.477	0.428	89.339
2019	*Nurses*	-	*0.468*	-	-
	GDP per capita rank	0.033	0.257	0.008	1.808
	Age dependency ratio	−0.256	−0.044	0.011	2.406
	Hospitalisations	0.810	0.493	0.399	85.296
2023	*Nurses*	-	*0.439*	-	-
	GDP per capita rank	0.070	0.255	0.018	4.064
	Age dependency ratio	−0.541	−0.044	0.024	5.398
	Hospitalisations	0.758	0.477	0.362	82.397

^1^ Elasticity is determined by multiplying the slope coefficient by the ratio of the average values of the independent variable to the dependent variable. ^2^ The concentration index is calculated using the fractional rank of GDP per capita, weighted by each district’s population. Concentration indices presented in italics indicate total inequality in the distribution of the health resource variable.

## Data Availability

All inequality measures were calculated by the author using source data from publicly available domains of the National Statistical Institute of the Republic of Bulgaria, available at: [https://www.nsi.bg/en/statistical-data/141/429; https://www.nsi.bg/en/statistical-data/206/651; https://www.nsi.bg/en/statistical-data/240/778; https://www.nsi.bg/en/statistical-data/241/781; https://www.nsi.bg/en/statistical-data/206/643; https://ncpha.government.bg/index/124-spravochnik-zdraveopazvane.html?lang=en] accessed on 5 January 2026.
